# Complement activation at the motor end-plates in amyotrophic lateral sclerosis

**DOI:** 10.1186/s12974-016-0538-2

**Published:** 2016-04-07

**Authors:** Nawal Bahia El Idrissi, Sanne Bosch, Valeria Ramaglia, Eleonora Aronica, Frank Baas, Dirk Troost

**Affiliations:** Department of Genome Analysis, Academic Medical Center, Amsterdam, 1105 AZ The Netherlands; Department of Neuropathology, Academic Medical Center, Amsterdam, 1105 AZ The Netherlands

**Keywords:** Amyotrophic lateral sclerosis, Motor end-plates, Complement, C1q, MAC, CD55, CD59

## Abstract

**Background:**

Amyotrophic lateral sclerosis (ALS) is a fatal progressive neurodegenerative disease with no available therapy. Components of the innate immune system are activated in the spinal cord and central nervous system of ALS patients. Studies in the SOD1^G93A^ mouse show deposition of C1q and C3/C3b at the motor end-plate before neurological symptoms are apparent, suggesting that complement activation precedes neurodegeneration in this model. To obtain a better understanding of the role of complement at the motor end-plates in human ALS pathology, we analyzed post-mortem tissue of ALS donors for complement activation and its regulators.

**Methods:**

Post-mortem intercostal muscle biopsies were collected at autopsy from ALS (*n* = 11) and control (*n* = 6) donors. The samples were analyzed for C1q, membrane attack complex (MAC), CD55, and CD59 on the motor end-plates, using immunofluorescence or immunohistochemistry.

**Results:**

Here, we show that complement activation products and regulators are deposited on the motor end-plates of ALS patients. C1q co-localized with neurofilament in the intercostal muscle of ALS donors and was absent in controls (*P* = 0.001). In addition, C1q was found deposited on the motor end-plates in the intercostal muscle. MAC was also found deposited on motor end-plates that were innervated by nerves in the intercostal muscle of ALS donors but not in controls (*P* = 0.001).

High levels of the regulators CD55 and CD59 were detected at the motor end-plates of ALS donors but not in controls, suggesting an attempt to counteract complement activation and prevent MAC deposition on the end-plates before they are lost.

**Conclusions:**

This study provides evidence that complement activation products are deposited on innervated motor end-plates in the intercostal muscle of ALS donors, indicating that complement activation may precede end-plate denervation in human ALS. This study adds to the understanding of ALS pathology in man and identifies complement as a potential modifier of the disease process.

**Electronic supplementary material:**

The online version of this article (doi:10.1186/s12974-016-0538-2) contains supplementary material, which is available to authorized users.

## Background

Amyotrophic lateral sclerosis (ALS) is the most common adult-onset motor neuron disease [1]. It is characterized by progressive loss of both upper and lower motor neurons, leading to muscle atrophy and eventually death [2]. Most ALS cases (90 %) are sporadic, while 10 % are familial. Many genes have been identified for familial ALS;C9orf72, FUS, TARDBP are the most frequently affected genes. Mutations in the gene encoding for the copper-zinc superoxide dismutase-1 (SOD-1) are found in about 10 % of familial cases of the disease. The transgenic SOD1^G93A^ rodent model recapitulates onset and progression of ALS.

The mechanisms leading to ALS are still unclear; both cell autonomous and non-cell autonomous mechanisms are involved [3–5]. A role of neuroinflammation [6–8] and early involvement of the neuromuscular junction in the SOD1^G93A^ rodent model has been suggested [9, 10]. The complement system has been also associated with neuroinflammation in the ALS rodent model [8, 11]. Complement is a key component of the innate immunity, but it can cause harm to tissue. Regulators of the complement system permit elimination of pathogens or dead cells without injuring the host. When this balance is disrupted, complement activation causes injury to the host and contributes to pathology in various diseases [12–14].

A role for complement in the pathogenesis of ALS in man is suggested by different researchers. Elevated concentrations of complement activation products in serum and cerebrospinal fluid were detected in ALS patients. In the spinal cord and motor cortex of patients with sporadic ALS, mRNA for C1q, C4 and protein levels of complement proteins C1q, C3, and membrane attack complex (MAC) were elevated [15]. In addition, C1q and C4 were upregulated in motor neurons in murine ALS models [16, 17], whereas C3 was upregulated in the anterior horn areas containing motor neuron degeneration [11]. Other studies have also shown upregulation of the major proinflammatory C5a receptor, during disease progression in mouse motor neurons [18]. SOD1^G93A^ rat treated with C5aR antagonist displayed a significant extension of survival time and a reduction in end-stage motor scores, suggesting an important role for complement in the disease progression [11]. Increased expression of complement components C1qB, C4, factors B, C3, and C5 and a decrease in the expression of regulators CD55 (regulator of C3) and CD59a (regulator of MAC) was detected in the lumbar spinal cord of SOD1^G93A^ mice [19].

We have previously shown that complement activation products C3/C3b and C1q were present at the motor end-plates of SOD1^G93A^ mice before the appearance of symptoms and remained detectable at the symptomatic stage, suggesting that complement activation precedes neurodegeneration and plays an early role in this model [20]. Early damage at the end-plates is in line with the “dying-back” mechanism. Retrograde degeneration is detected in ALS patients [21, 22], and in transgenic SOD1^G93A^ mice, retraction of motor axons from their muscle synapse has been shown to occur before any symptoms of the disease appear in the muscle [23], suggesting the disease starts at the motor end-plates.

Here, we analyzed whether key complement components and regulators are also deposited at the motor end-plates in post-mortem intercostal muscles of human ALS cases. We tested for the presence of complement components C1q and MAC and for the regulators CD55 and CD59 in this tissue.

## Methods

### Ethics statements

Tissue was obtained and used in accordance with the Declaration of Helsinki and the Academic Medical Center Research Code provided by the Medical Ethics Committee. Informed consent was obtained from all the patients.

### Tissue processing human intercostal muscle

Post-mortem intercostal muscle biopsies were collected at autopsy from sex-matched ALS (*n* = 11) and control (*n* = 6) donors at the Department of Neuropathology of the Academic Medical Center (University of Amsterdam). All cases were reviewed by a neuropathologist and diagnosed according to the standard histopathological criteria. None of our patients were on respiratory support. Muscle samples were snap-frozen in liquid nitrogen and stored at −80 °C until processed. Detailed information about sex, age, and clinical features of ALS and control donors are given in Tables 1 and 2. In this study, we included material from ALS donors with familial and sporadic ALS. We used age-matched controls, which did not suffer from neuromuscular or neurological disease. The tissue was subsequently embedded in Tissue-Tek, optimal cutting temperature compound (OCT) (Sakura, Zoeterwoude, NL) and cut using cryostat (Reichert Jung; Leica, Nussloch, Germany); cryosections of 6 and 40-μm were cut and stored at −80 °C until immuno- and fluorescence stainings were performed.

**Table 1:** Demographic and clinical data of ALS donors

Patient nr	Gender	Age of onset	PMD (hours)	Disease duration (years)	ALS type
1	F	66	6	4.5	Sporadic ALS
2	F	61	Unknown	1	Sporadic ALS
3	M	56	10	3.5	Sporadic ALS
4	F	80	Unknown	2	Sporadic ALS
5	M	68	Unknown	3.5	Familial ALS^a^
6	F	57	9.5	3.5	Sporadic ALS
7	M	62	Unknown	4	Sporadic ALS
8	M	72	Unknown	X	Sporadic ALS
9	M	68	Unknown	3.5	Sporadic ALS
10	M	54	Unknown	1.5	Familial ALS^a^
11	F	58	10–11	2	Sporadic ALS

All the cases analyzed show classical pathology with motor cell loss and degeneration of the corticospinal tracts, including P62 and phosphorylated TDP43 inclusions in motor cells

*PMD* post-mortem delay

^a^C9ORF repeat

**Table 2:** Demographic and clinical data control donors

Patient nr	Gender	Age	PMD (hours)
1	M	69	5–6
2	F	65	Unknown
3	F	55	3.5–4
4	M	73	11
5	M	62	4–8
6	F	68	10

### Nonspecific esterase reaction followed by immunostaining

Fresh frozen sections of 6 μm were tested for the nonspecific esterase (NE) reaction according to the technique of Lehrer and Ornstein [24]. After the NE staining, the sections were fixed for 10 min in 4 % paraformaldehyde (PFA) and after washed in PBS. The sections were permeabalized in PBS/0.2 % TritonX and blocked for 1 h at room temperature (RT) using PBS/5 % fetal calf serum (FCS)/0.2 % TritonX (blockmix). The primary antibodies anti-C5b-9 for MAC recognizes a neo-epitope in C9 (aE11 clone, Dako, Carpinteria, CA), anti-C1q (Dako, F 0254, Denmark), or anti-decay-accelerating factor (anti-DAF) (Abcam, Ab20145, Cambridge, MA, USA) were diluted in blockmix according to Table 3 and incubated for 1 h at room temperature. The sections were washed with PBS three times and incubated with the secondary antibody PowerVision poly-AP anti-mouse IgG or poly-AP anti-rabbit IgG (Immunologic, DPVM55A, Netherlands) for 45 min. After washing, the sections were developed with VECTOR Blue Alkaline Phosphatase (AP) Substrate Kit (SK-5300). The sections were air-dried and mounted using VectaMount (Vector laboratories, H-5000-60, USA).

**Table 3:** Primary antibodies and their dilutions

Antigen	Species	Specificity	Type	Dilution	Art.#/company
Neurofilament heavy chain (NF-H)	Rabbit polyclonal	Anti-neurofilament	Anti-human	1:1000	ab8135/Abcam
MAC (ae11 clone)	Mouse monoclonal	Anti-C5b-9 complex	Anti-human	1:200	M0777/Dako
FITC-conjugated C1q complement	Polyclonal rabbit	Anti-C1q	Anti-human	1:50	F0254/Dako
FITC-conjugated DAF	Mouse monoclonal	Anti-CD55	Anti-human	1:40	555693/BD Pharmigen
CD59	Mouse monoclonal	Anti-CD59	Anti-human	1:50	HM2120/Hycult
C1q	Polyclonal rabbit	Anti-C1q	Anti-human	1:50	A0136/Dako
FITC-conjugated C3c complement	Polyclonal rabbit	Anti-C3	Anti-human	1:50	ab4212/Abcam
DAF	Mouse monoclonal	Anti-CD55	Anti-human	1:150	Ab20145/Abcam
Synaptophysin	Rabbit monoclonal	Anti-synaptophysin	Anti-human	1:50	RM-9111-S/Thermo Scientific
Synaptophysin	Mouse monoclonal	Anti-synaptophysin	Anti-human	1:200	M0776/Dako
S100b	Rabbit polyclonal	Anti-S100b	Anti-human	1:100	Z0311/Dako
S100b	Mouse monoclonal	Anti-S100b	Anti-human	1:500	S2532/Sigma

### Animals

SOD1^G93A^ transgenic ALS mice [high copy number; B6SJLTg (SOD1-G93A)1Gur/J] (Gurney, 1994b) and wild-type (B6SJL) littermates were housed in groups at 20 °C on 12:12 h light-dark cycle, with free access to food and water. Experimental protocols complied with national animal care guidelines, licensed by the responsible authority. All animals were free of microbiological infection (FELASA screened).

### Tissue processing SOD^G93A^ and wild-type mice

SOD1^G93A^ and wild-type mice were sacrificed at post-natal day 47 (presymptomatic stage SOD1^G93A^*n* = 4; wild-type *n* = 4) by CO_2_ inhalation. The gastrocnemius muscle was dissected, post-fixed overnight in 4 % paraformaldehyde/PBS at 4 °C, cryoprotected in 30 % sucrose/PBS for 72 h at 4 °C, and embedded in Tissue-TEK OTC; cryosections of 40-μm were cut and stored at −80 °C until used for histology.

### Immunofluorescence staining

For immunofluorescence staining of SOD1^G93A^ gastrocnemius muscle and human intercostal muscle tissue, sections were air-dried and fixed for 10 min in 4 % PFA at −20 °C. Slides were washed in PBS and permeabalized in PBS/0.2 % TritonX. Subsequently, sections were blocked for 1 h at room temperature (RT) using PBS/5 % FCS/0.2 % TritonX (blockmix). Primary antibodies were diluted in blockmix according to Table 3 and incubated overnight at 4 °C. The following primary antibodies were used (see Table 3 for specifications): anti-neurofilament heavy-chain (NF-H, Abcam, Cambridge UK) nerves, anti-synaptophysin detecting the motor nerve terminal (RM-911-S/Thermo Scientific, USA, or M0776/Dako, Carpinteria, CA), anti-S100b recognizing the terminal Schwann cells (Z0311/Dako, Carpinteria, CA or S2532/Sigma, USA), anti-C5b-9 for MAC (aE11 clone, Dako, Carpinteria, CA), anti-C1q-FITC conjugated (DAKO, Denmark), anti-C3c-FITC conjugated recognizing C3c part of C3 and C3b, anti-DAF-FITC conjugated detecting the regulator of complement CD55 (BD Pharmingen), and anti-CD59 detecting the regulator of MAC (Hycult Biotech). The sections were washed in PBS, and secondary antibodies was applied, diluted according to Table 4 in blockmix, and incubated for 2 h at RT. Used secondary antibodies are anti-mouse FITC (Jackson Immunoresearch, West Grove, PA), anti-rabbit and anti-mouse Cy3 (Jackson Immuno Research, West Grove, PA), and anti-mouse Cy5 (Invitrogen, Germany). After washing off the secondary antibody, α-bungarotoxin (α-BTX)-Alexa 488 conjugate (Molecular Probes) (1:500), which binds to post-synaptic acetylcholine receptors on the muscle fibers, was applied for 20 min at room temperature to the sections to visualize the end-plates. The sections then were washed in PBS and air-dried. Vectashield medium (Vector Laboratories Inc, Burlingame, USA) was used for mounting.

**Table 4:** Secondary antibodies, dilutions, and excitation/emission rate

Fluorochrome	Specificity	Dilution	Art.#/company	Excitation/emission
FITC	Anti-mouse	1:150	Jackson Immuno Research/200-542-037	493/519
Cy3	Anti-mouse	1:150	Jackson Immuno Research/200-162-037	550/570
Cy3	Anti-rabbit	1:150	Jackson Immuno Research/711-165-152	550/570
Cy5	Anti-mouse	1:450	Invitrogen/A10524	649/665
Alexa 488-α-BTX	Snake	1:500	Anti-nicotinic acetylcholinereceptor	495/519

### Microscopy

The 40-μm muscle sections were analyzed for the positivity for fluorophores Cy3 (550–570 nm), Cy5 (649/665 nm), and alpha-bungarotoxin-Alexa 488 (488–520 nm) using a Leica TCS SP8 X confocal microscope (Leica Microsystems B.V., Rijswijk, The Netherlands). For each view, Z-stacks (objective ×40/1.30 oil; 290 μm × 290 μm) of 40-μm thick muscle tissue were made. The images were analyzed using Leica LCS software (Leica).

### Quantification

For each view, Z-stacks of 40-μm thick muscle were examined and scored on the total number of immunoreactivity for Alexa 488-α-BTX/MAC, Alexa 488-α-BTX/CD59-positive end-plates as well as the total number of NF-H staining co-localizing with specific complement antibodies C1q and CD55 (Table 3). For each muscle sample of an individual donor, 20 non-overlapping microscopic views of 40-μm thick sections were examined (total volume 6.73 × 10^7^ μm^3^).

Each motor end-plate identified with Alexa 488-α-BTX on the surface of a muscle fiber was counted, and the length of the end-plates were measured in both ALS and control muscle biopsies. The size of end-plates was measured using Leica application suite X software (LASX software, Microsystems B.V., Rijswijk, The Netherlands) 3D visualizer, excluding the end-plates that were not completely in the 3D image. The number of immunoreactive area per section was scored and expressed as standard deviation of the mean (SD).

### Statistical analysis

Data analysis was performed using GraphPad Prism version 5.0 (GraphPad Software Inc, San Diego, CA, USA) statistical package. Student’s *t* test was performed for statistical analyses comparing two groups. For comparison of more than two groups, one-way ANOVA with Bonferroni multiple comparison post hoc test was used when the data was normally distributed. For non-normally distributed data, the Kruskal-Wallis test was used. Differences were considered statistically significant when *P* ≤ 0.05.

## Results

### Motor end-plates in ALS intercostal muscle

**Fig. 1:**
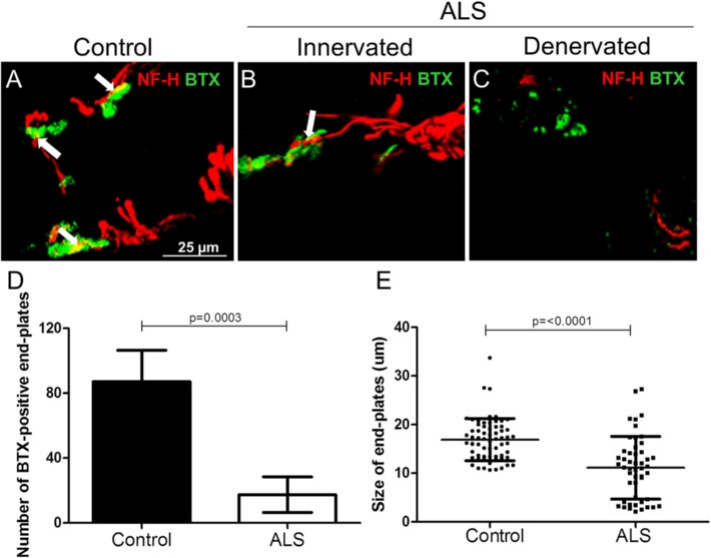
Number and size of α-BTX-positive end-plates in intercostal muscle of ALS donors. Confocal microscopic images of motor end-plates from controls (**a**) and ALS donors (**b**, **c**) double-labeled with α-bungarotoxin (α-BTX, Alexa 488) and antibodies against neurofilament (NF-H, Cy3). All controls showed co-localization of NF-H with α-BTX (*white arrows*). In ALS, both innervated end-plates (panel **b**) and denervated end-plates (panel **c**) were detected. The number and size of α-BTX-positive end-plates in 20 non-overlapping Z-stacks in 40-μm thick intercostal muscle sections is shown in panels **d** and **e**. Both number and size of α-BTX-positive end-plates of ALS donors (*n* = 2) are reduced compared to controls (*n* = 2) (*P* = 0.0003 and *P* = <0.0001, respectively). *Error bars* represent standard deviation of the mean

**Fig. 2:**
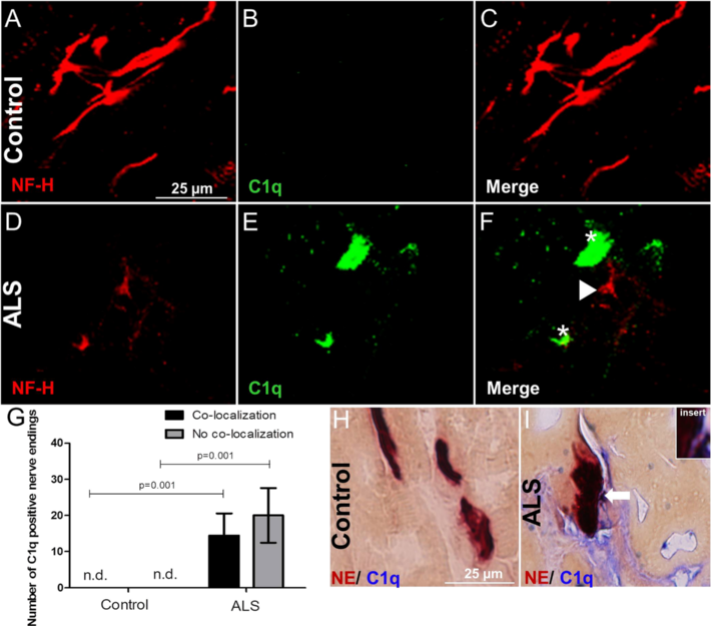
Confocal microscopic images of intercostal muscle from controls (**a**, **b**, **c**) and ALS donors (**d**, **e**, **f**) double-labeled with antibodies against neurofilament (NF-H, Cy3) and antibodies against classical pathway component of the complement system C1q (C1q, FITC). C1q deposition was detected on the nerves as well as near the nerve endings (*white asterisks* in **f**) in muscle of ALS donors but not in controls. **g** Quantification showed C1q-positive staining co-localizing with nerves and in the vicinity of nerve endings (*white arrowhead* pointing to NF-H and *asterisk* on C1q in **f**) in the intercostal muscle of ALS donors but not in controls (*P* = 0.001 and *P* = 0.001, respectively). NE staining (*dark brown*) followed by an immune staining for C1q (*blue*) showed (**i**) C1q deposition on the end-plates of ALS donors (*white arrow* in **i** and enlargement of the area as *insert*) (**h**) by contrast, no C1q deposition was found deposited on the motor end-plates in the intercostal muscle of control donors. Numbers of C1q-positive nerve endings in 20 non-overlapping Z-stacks in 40-μm thick intercostal muscle sections is given on the *y*-axis. *Error bars* represent standard deviation of the mean. *n.d.* not detected

The size and number of motor end-plates were analyzed in the intercostal muscle of ALS donors and age- and sex-matched controls. For analysis of both nerves and motor end-plates, confocal microscopy was performed on 40-μm thick intercostal muscle sections of ALS and control donors that were stained for Alexa 448 α-BTX detecting the motor end-plates and neurofilament heavy-chain antibody (NF-H). Alexa 448 α-BTX positive and negative end-plates were expected in the intercostal muscle of ALS post-mortem tissue given that the average age of the ALS donors was 64 years and that failure of the respiratory muscle occurs in the end-stage of the disease in these patients. All control muscles showed co-localization of α-BTX and NF-H. The BTX-positive end-plates were divided in two groups: (1) end-plates co-localizing with NF-H (innervated) (Fig. 1a, b) and (2) end-plates that showed no co-localization with NF-H (denervated) (Fig. 1c).

The average number of α-BTX-positive end-plates in the intercostal muscles were 87 in controls and 17 in ALS donors per 20 non-overlapping microscopic views. Thus, the intercostal muscle of ALS donors showed a significantly lower number of α-BTX-positive motor end-plates (*P* = 0.0003) (Fig. 1d). The percentage of innervated and denervated end-plates were 30 and 70 %, respectively, whereas the control donors show 100 % innervation. We also analyzed the size of the α-BTX-positive end-plates in the intercostal muscle of two ALS patients and two age-matched controls. The controls showed a mean of 16.9 μm [SD 4,34) (*n* = 2), and the ALS cases showed a mean of 11.10 μm [SD 6,44) (*P* = <0.0001) (*n* = 2) (Fig. 1e). All end-plates in the 20 non-overlapping views were counted.

### C1q deposition on the motor end-plates in the intercostal muscle of ALS donors

C1q deposits were detected before the appearance of clinical symptoms at the muscle end-plate of the SOD1^G93A^ mouse model [20]. This suggests that complement activation is an early event. Here, we tested in an overview experiment whether C1q deposits are also present in the muscle of ALS donors and if C1q is specifically deposited on the motor end-plate. Immunofluorescence for NF-H and C1q was performed on the intercostal muscle of control (Fig. 2a, b, c) and ALS (Fig. 2d, e, f) donors. For each individual, we analyzed 20 non-overlapping Z-stacks in 40-μm thick sections using confocal microscopy. C1q immunoreactivity was present in the majority of the intercostal muscle tissue of ALS donors (Fig. 2d, e, f). An average of 14 [control vs ALS *P* = 0.001) of the C1q immunoreactive regions in the intercostal muscle of ALS donors were co-localizing with NF-H staining (Fig. 2g, black bar). An additional 20 areas [control versus ALS *P* = 0.001) of the C1q immunoreactivity were found in the vicinity of NF-H staining (Fig. 2g, gray bar). No C1q immunoreactivity was detected in the intercostal muscle of age-matched controls (Fig. 2b). The NF-H immunoreactivity was generally stronger in the intercostal muscles of control compared to ALS donors (data not shown).

To determine whether C1q is deposited on the end-plates, we performed a NE staining on frozen intercostal muscles of control and ALS donors to visualize the end-plates followed by an immunostaining for C1q. The immunostaining showed an extensive amount of C1q deposited on and around the end-plates of ALS donors (Fig. 2i). No C1q deposition was detected in on the end-plates of control donors (Fig. 2h). We also detected C1q on the cellular elements synaptophysin and S100b indicating C1q is also deposited at the motor nerve terminal and terminal Schwann cell in the intercostal muscle of ALS donors (Additional file 1: Figure S1B, D, arrows) but not in controls (Additional file 1: Figure S1A, C).

### MAC deposition on the motor end-plates in the intercostal muscle of ALS donors

**Fig. 3:**
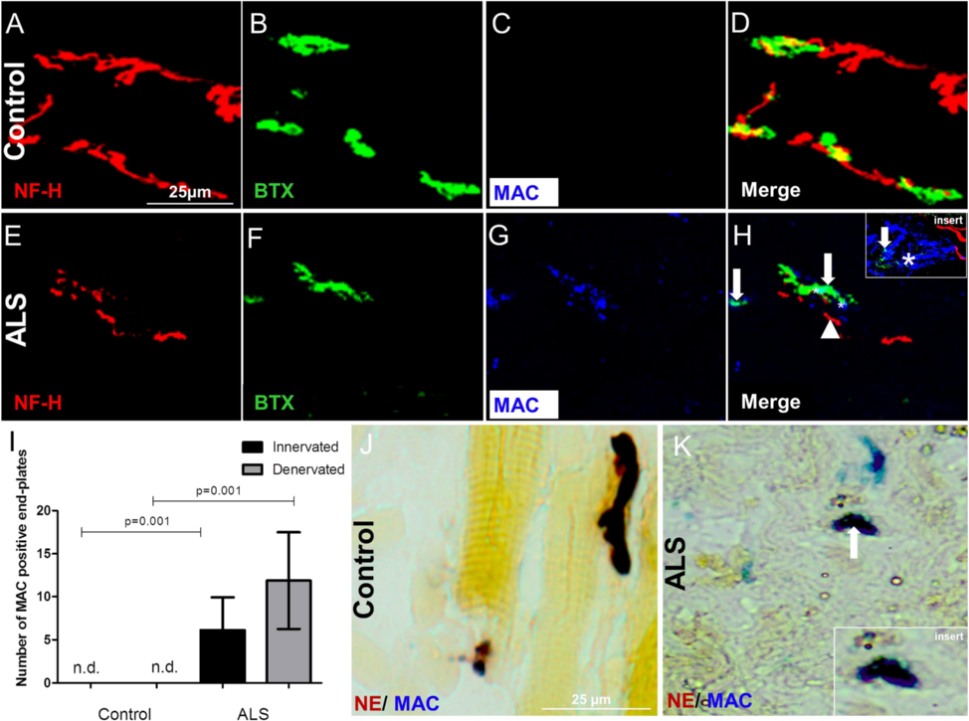
Representative confocal images of triple-immunofluorescence staining for neurofilament (NF-H, Cy3), motor end-plates with α-BTX (Alexa 488), and complement component C5b-9 with MAC (Cy5) in control (**a**, **b**, **c**, **d**) and ALS intercostal muscle (**e**, **f**, **g**, **h**), shows presence of MAC (*white asterisks* in **h** and enlarged in the *insert*) on end-plates (*white arrows* in **h**) and around nerves in ALS muscle (*white arrowhead* in **h**) but not in controls (**c**, **d**). Quantification showed a significantly higher percentage of MAC-positive innervated end-plates (*P* = 0.001) and denervated end-plates (*P* = 0.001) in ALS intercostal muscle compared to controls. Numbers of MAC-positive end-plates in 20 non-overlapping Z-stacks in 40-μm thick intercostal muscle sections is given on the *y*-axis. *Error bars* represent standard deviation of the mean (**i**). NE staining (*dark brown*) followed by an immune staining for MAC (*blue*) showed (**k**) MAC deposition deposited on the end-plates of ALS donors (*white arrow* in **k** enlarged in the *insert*), **j** but not on end-plates of control donors. *n.d.* not detected

To determine whether the terminal pathway of the complement system is also activated in ALS, we tested for MAC deposition at the motor end-plates. We analyzed the intercostal muscle of ALS donors. The presence of MAC on innervated or denervated motor end-plates was measured using immunofluorescence and confocal microscopy on 40-μm thick sections. We analyzed 20 non-overlapping Z-stacks. Human intercostal muscles of control (Fig. 3a, b, c, d) and ALS donors (Fig. 3e, f, g, h) were stained for NF-H, α-BTX detecting end-plates, and C9neo epitope, a component of the terminal complement complex MAC (C5b9). MAC immunoreactivity was detected on and around nerves and on motor end-plates in ALS patients (Fig. 3e, f, g, h). A strong MAC immunoreactiviy was detected (Fig. 3h, asterisks within insert) on the end-plates with a weak α-BTX immunoreactivity (Fig. 3h, arrow within insert). By contrast, a weak MAC immunoreactivity (Fig. 3h, asterisks) was detected on end-plates with strong α-BTX immunoreactivity (Fig. 3h, arrow) and nerves innervating the motor end-plate (Fig. 3h, arrow head). We suggest there might be a relevant anti-correlation between MAC and α-BTX immunoreactivity in the ALS samples. However, the high variability between the biological specimens and the low number of end-plates detected in these samples make it difficult to draw firm conclusions based on the measurement of fluorescence intensities.

No MAC immunoreactivity was detected on or around the end-plates of control donors (Fig. 3c, d). Quantification showed a mean of six innervated (controls vs ALS donors *P* = 0.01) and 11 denervated (control versus ALS donors *P* = 0.01) MAC-positive motor end-plates in 20 non-overlapping Z-stacks in 40-μm thick intercostal muscle sections (Fig. 3i).

To determine whether MAC is deposited on the motor end-plates, we performed immunostainings for MAC followed by NE staining on the intercostal muscle of control (Fig. 3j) and ALS donors (Fig. 3k). We found MAC deposition on the motor end-plates in the intercostal muscle of ALS donors but not in controls, suggesting that the terminal pathway of the complement system is activated on the motor end-plates. We also detected MAC on the cellular elements synaptophysin and S100b indicating that MAC is also deposited at the motor nerve terminal and terminal Schwann cell in the intercostal muscle of ALS donors (Additional file 2: Figure S2B, D, arrows) but not in controls (Additional file 2: Figure S2A, C).

### CD55 on the motor end-plates in the intercostal muscle of ALS donors

**Fig. 4:**
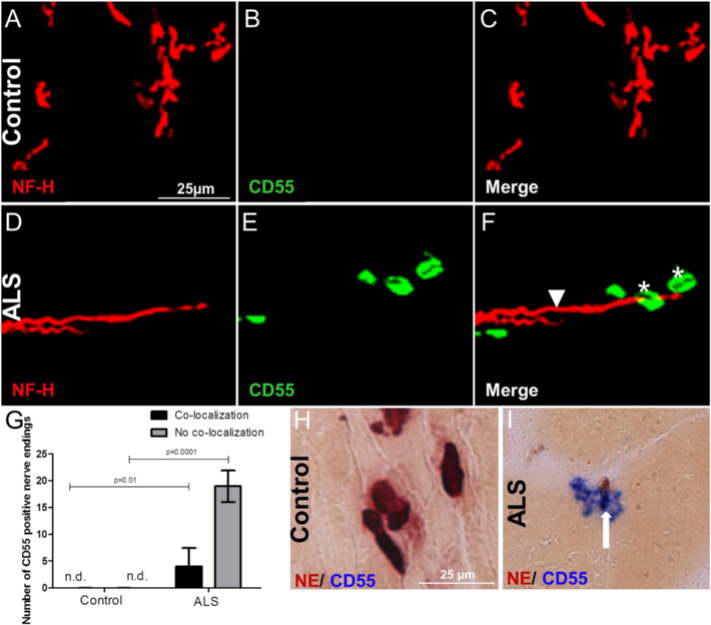
Representative confocal double-immunofluorescence for neurofilament (NF-H, Cy3) and CD55 detected with anti-DAF (FITC) in control (**a**, **b**, **c**) and ALS (**d**, **e**, **f**) intercostal muscle shows CD55 deposition in ALS intercostal muscle on and around nerves (*white asterisks* on CD55 and *arrowhead* pointing to NF-H in **f**) but not in control tissue (**c**). Quantification showed CD55 deposition co-localizing with nerves or in the vicinity of nerves in the intercostal muscle of ALS donors but not in controls (*P* = 0.01 and *P* = 0.0001, respectively) (**g**). Numbers of CD55-positive end-plates in 20 non-overlapping Z-stacks in 40-μm thick intercostal muscle sections is given on the *y*-axis. *Error bars* represent standard deviation of the mean *n.d.* not detected. NE staining (*dark brown*) followed by an immune staining for CD55 (*blue*) showing **i** CD55 deposition on the motor end-plates (*white arrow* in **i**) in the intercostal muscle of ALS donors **h** but no CD55 deposition in controls

Regulators such as CD55 and CD59 protect tissues against an attack by the complement system. The role of these regulators in the pathogenesis in ALS is of interest. CD55 acts on the membranes of self-cells to circumvent the deposition of C3b on their surfaces [25]. We found C3/C3b deposition in the intercostal muscle of ALS donors deposited at the motor nerve terminal and terminal Schwann cells (Additional file 3: Figure S3B, D, arrows) but not in controls (Additional file 3: Figure S3A, C). Therefore, we analyzed whether CD55 is also deposited in the intercostal muscle ALS donors. We analyzed 20 non-overlapping Z-stacks in 40-μm thick sections using confocal microscopy. Human intercostal muscles of control (Fig. 4a, b, c) and ALS donors (Fig. 4d, e, f) were stained for NF-H and CD55. We identified strong staining for CD55 on and around nerves in the intercostal muscle of ALS donors (Fig. 4f) but not in controls (Fig. 4c). Quantification showed a significantly higher percentage of CD55-positive staining. Not all staining co-localized with NF-H in the intercostal muscle of ALS donors (Fig. 4g, gray bar).

To determine whether CD55 is deposited on the end-plates, a NE staining on frozen intercostal muscle of control and ALS donors was performed to visualize the end-plates followed by immunostaining for CD55. No CD55 deposition was detected on the end-plates of control donors (Fig. 4c, h), by contrast an extensive amount of CD55 was found deposited on and around the end-plates of ALS donors (Fig. 4i), suggesting an increased regulation of the common complement pathway on the end-plates. We also detected CD55 on the cellular elements synaptophysin and S100b indicating that CD55 is also deposited at the motor nerve terminal and terminal Schwann cell in the intercostal muscle of ALS donors (Additional file 4: Figure S4B, D, arrows) but not in controls (Additional file 4: Figure S4A, C).

### CD59 on the motor end-plates in the intercostal muscle of ALS donor

**Fig. 5:**
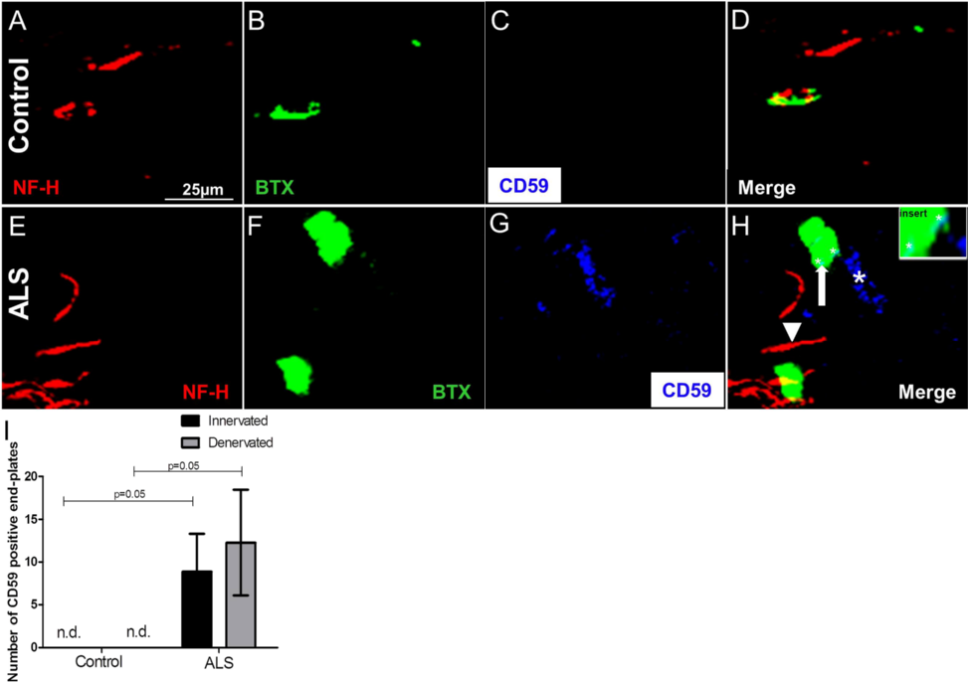
Representative confocal triple-immunofluorescence for neurofilament (NF-H, Cy3), end-plates detected with α-BTX (Alexa 488) and the regulator CD59 (Cy5) in control (**a**, **b**, **c**, **d**) and ALS (**e**, **f**, **g**, **h**) intercostal muscle showing deposition of CD59 (*white asterisks* in **h**, enlarged in *insert*) in ALS intercostal muscle tissue on denervated end-plates (*white arrow* pointing to α-BTX and *arrowhead* pointing to NF-H in **h**) but not in controls. Quantification shows CD59-positive innervated and denervated motor end-plates in the intercostal muscle of ALS donors but not in controls (*P* = 0.05 and *P* = 0.05, respectively) (**i**). Data represents standard deviation of the mean. *n.d.* not detected

The glycolipid anchored protein CD59 has a binding site for both C8 and C9 and as such can prevent formation of MAC [26, 27]. Immunofluorescence staining for NF-H, α-BTX detecting end-plates, and the regulator CD59 was performed on intercostal muscle of control (Fig. 5a–d) and ALS (Fig. 5e–h) donors. We analyzed 20 non-overlapping Z-stacks in 40-μm thick sections using confocal microscopy. CD59 was found abundantly present on and around the motor end-plates in the intercostal muscle of ALS donors (Fig. 5g, h, asterisks) but was negative in the intercostal muscle of control donors (Fig. 5c, d). Quantification showed that this difference is significant for both innervated and denervated motor end-plates of ALS donors (Fig. 5i) (*P* = 0.05, *P* = 0.05, respectively). In addition, we show that CD59 is also deposited on the motor nerve terminal and terminal Schwann cells in the intercostal muscle of ALS donors (Additional file 5: Figure S5B, D, arrows) but not in controls (Additional file 5: Figure S5A, C).

## Discussion

Although a role for complement has been found in many neurodegenerative diseases [28–34], its contribution to disease progression in animal models for ALS is controversial [35, 36]. We previously provided evidence for an early role of the complement system in the SOD1^G93A^ mouse model of familial ALS [20]. Fischer and colleagues suggest that ALS pathology starts at the muscle end-plates proceeding to the spinal cord and subsequently the brain [23]. In addition, several physiological and morphological alterations have been reported on the muscle end-plates from in vivo and ex vivo mouse and rat preparations [37–43].

To obtain a better understanding of the role of complement in human ALS pathology, we analyzed post-mortem tissue of ALS donors for complement activation products and its regulators. We found a lower number and a decreased size of the α-BTX-positive end-plates in the tissue of ALS donors compared to controls, suggesting that the end-plates in the intercostal muscle of ALS patients are affected.

In the ALS muscle, we found deposition of complement activation products C1q and C3 but not in controls. C1q and C3 were detected not only on and around the end-plates but also on the nerve terminal and terminal Schwann cells. C1q and C3 mRNA and protein levels were found elevated in spinal cord and motor cortex of patients with sporadic ALS [15]. In murine ALS models, C1q was also upregulated in motor neurons [16], whereas C3 is upregulated in the anterior horn areas containing motor neuron degeneration. Expression profiling in the mutant SOD1 motor neurons showed that C1q genes were upregulated early in the disease. C1q can bind antibody aggregates and activate the classic complement pathway [11, 17]. This data suggests a role for C1q and C3 in ALS. However, a study by Lobsiger et al. demonstrates no significant pathogenic role for C1q and C3 proteins in the survival of SOD1^G93A^ ALS mice, contradicting a possible role for complement in this model [44]. This study, however, did not analyze downstream pathways, like the extrinsic pathway of complement which can lead to C5 cleavage and does not need C1q and C3 proteins for activation and MAC formation, which may be the key point at which complement-mediated neurotoxicity occurs in these ALS models [35, 45].

A role for MAC in the pathology of neurological disorders is suggested, including ALS [31]. In serum of ALS patients, the terminal complement activation products C5a and MAC are elevated [46]. MAC can damage tissue and target nerves in different neurodegenerative models [34, 47], suggesting a role for MAC in degeneration. Furthermore, we show that MAC is deposited on the motor end-plates on day 47 in the SOD1^G93A^ mouse model, suggesting that MAC deposition is an early event in this model (Additional file 6: Figure S6). This result is in contrast to a previous analysis of the SOD^G93A^ mice [20]. In that study, no MAC was detected on the end-plates of the SOD^G93A^ mice. We attribute this difference to the use of another antibody for the detection of C5b9. We used a monoclonal mouse anti-human C9neo, this antibody gives a specific signal on both frozen human and mouse sections. It detects C9neo and not C9; therefore, it is also more specific to recognize the C9 within the MAC, whereas the polyclonal mouse anti-rat C9 that was used previously either gives no staining or a lot of background on frozen sections. Since we find MAC deposition consistently on end-plates in both human and mouse muscle, an artifact is excluded.

The present study shows deposition of MAC at the muscle end-plates of ALS donors. We show strong MAC immunoreactiviy on the end-plates with a weak α-BTX immunoreactivity in the intercostal muscle of ALS donors. By contrast, a weak MAC immunoreactivity was detected on end-plates with strong α-BTX immunoreactivity. This is compatible with a model in which MAC deposition occurs before loss of the end-plates, in fact MAC could be a contributor to disease progression and end-plate pathology. In addition, MAC was also found co-localizing with the motor nerve terminal and terminal Schwann cells.

The propensity of the MAC to “drift” from the site of activation and deposit on other sites may even result in more damage to the muscle. In general, cells are protected from complement attack by multiple complement regulators, preventing damage. This protection can be overwhelmed resulting in damage to tissue and drives inflammation [48, 49].

CD55 and CD59 restrict complement activation by inhibiting C3/C5 convertase activities and membrane attack complex formation, respectively. In the actively immunized experimental autoimmune myasthenia gravis mice deficient in either CD55 or CD59, a significant increase in complement deposition at the end-plates was observed and worsened disease outcome associated with increased levels of serum cytokines was observed [50].

We demonstrate that also the regulators of the common pathway CD55 and the terminal pathway CD59 are deposited on the motor end-plates of ALS donors, but not in controls. In addition, the motor nerve terminal and terminal Schwann cells were also co-localizing with CD55 and CD59. Upregulation of the complement regulators CD55 and CD59 on the motor end-plates of ALS patients probably is an attempt to dampen the high level of complement activation and protect the tissue.

Since we also detected MAC deposition at the motor end-plates of the ALS donors, the upregulation of CD55 and CD59 is not sufficient to protect the end-plates from MAC attack.

## Conclusions

In summary, we demonstrated that complement activation products C1q and MAC are deposited on motor end-plates in post-mortem tissue of ALS donors. MAC was found deposited on motor end-plates that were innervated by nerves, indicating that complement activation may precede motor end-plate denervation.

Here, we showed that the regulators CD55 and CD59 are also expressed on the motor end-plates, indicating an attempt to control the activation. This process is probably not efficient enough because MAC can still be detected on the α-BTX-positive motor end-plates. Since a role for MAC in the pathology of neurological disorders is suggested [31], detecting complement deposited at the end-plates of ALS donors, before the end-plates are lost, suggests that complement is an early event in ALS and might play an important role in the motor end-plate pathology in ALS. This observation is in line with earlier studies suggesting a dying-back mechanism in ALS, meaning the disease probably starts at the motor end-plates [23]. Although this study was performed using post-mortem intercostal muscle tissue of ALS patients and there may be some limitations to our conclusions about complement being involved in motor end-plate degeneration, this study adds to the understanding of ALS pathology in man.

## Additional files

Additional file 1: Figure S1. Representative confocal immunofluorescence for synaptophysin (SYN-Cy3) detecting the motor nerve terminal (A, B) or S100b (Cy3) detecting the terminal Schwann cells (C, D) double stained with anti-C1q (FITC) in control (A, C) and ALS (B, D) intercostal muscle shows C1q co-localizing with both synaptophysin and S100b (white arrow in B and D, respectively) but no C1q deposition in controls. (TIF 1461 kb) Additional file 2: Figure S2. Representative confocal immunofluorescence for synaptophysin (SYN-Cy3) detecting the motor nerve terminal (A, B) or S100b (Cy3) detecting the terminal Schwann cells (C, D) double stained with an antibody detecting MAC (FITC) in control (A, C) and ALS (B, D) intercostal muscle shows MAC deposition on both the motor nerve terminal and the terminal Schwann cells (white arrow in B and D, respectively) but no MAC deposition in controls. (TIF 2178 kb) Additional file 3: Figure S3. Representative confocal immunofluorescence for synaptophysin (SYN-Cy3) detecting the motor nerve terminal (A, B) or S100b (Cy3) detecting the terminal Schwann cells (C, D) double stained with anti-C3c recognizing C3c part of C3 and C3b (FITC) in control (A, C) and ALS (B, D) intercostal muscle shows C3c co-localizing with both synaptophysin and S100b (white arrow in B and D, respectively), but no C3c deposition in controls. (TIF 1624 kb) Additional file 4: Figure S4. Representative confocal immunofluorescence for synaptophysin (SYN-Cy3) detecting the motor nerve terminal (A, B) or S100b (Cy3) detecting the terminal Schwann cells (C, D) double stained with anti-CD55 (FITC) in control (A, C) and ALS (B, D) intercostal muscle shows CD55 co-localizing with both synaptophysin and S100b (white arrow in B and D, respectively) but no CD55 deposition in controls. (TIF 1271 kb) Additional file 5: Figure S5. Representative confocal immunofluorescence for synaptophysin (SYN-Cy3) detecting the motor nerve terminal (A, B) or S100b (Cy3) detecting the terminal Schwann cells (C, D) double stained with anti-CD59 (FITC) in control (A, C) and ALS (B, D) intercostal muscle shows CD59 deposition on both the motor nerve terminal and the terminal Schwann cells (white arrow in B and D, respectively) but no CD59 deposition in controls. (TIF 1202 kb) Additional file 6: Figure S6. Representative confocal microscopy images of the motor end-plate from wild-type (*n* = 4) (A) and SOD1^G93A^ mice (*n* = 4) at 47 (B), immunostained for neurofilament NF-H (white arrow head in A and B), MAC with C5b9 (white asterisk in B), and the muscle end-plate with α-BTX (Alexa 488), showing deposition of MAC (white asterisk in B) on the innervated motor end-plate (white arrow pointing to NF-H co-localizing with α-BTX) in SOD1^G93A^ mice but not in the wild-type mice. Bar = 20 μm. (TIF 869 kb)

## Abbreviations

ALS: amyotrophic lateral sclerosis
MAC: membrane attack complex
NE: nonspecific esterase
NF-H: neurofilament heavy-chain antibody
SOD-1: copper-zinc superoxide dismutase-1
α-BTX: α-bungarotoxin

## References

[1] Pasinelli P, Brown RH (2006). Molecular biology of amyotrophic lateral sclerosis: insights from genetics. Nat Rev Neurosci.

[2] Mitchell JD, Borasio GD (2007). Amyotrophic lateral sclerosis. Lancet.

[3] Raoul C, Estevez AG, Nishimune H (2002). Motoneuron death triggered by a specific pathway downstream of Fas. potentiation by ALS-linked SOD1 mutations. Neuron.

[4] Boillee S, Yamanaka K, Lobsiger CS (2006). Onset and progression in inherited ALS determined by motor neurons and microglia. Science.

[5] Di Giorgio FP, Carrasco MA, Siao MC, Maniatis T, Eggan K (2007). Non-cell autonomous effect of glia on motor neurons in an embryonic stem cell-based ALS model. Nat Neurosci.

[6] Bruijn LI, Miller TM, Cleveland DW (2004). Unraveling the mechanisms involved in motor neuron degeneration in ALS. Annu Rev Neurosci.

[7] Cozzolino M, Ferri A, Carri MT (2008). Amyotrophic lateral sclerosis: from current developments in the laboratory to clinical implications. Antioxid Redox Signal.

[8] Woodruff TM, Costantini KJ, Taylor SM, Noakes PG (2008). Role of complement in motor neuron disease: animal models and therapeutic potential of complement inhibitors. Adv Exp Med Biol.

[9] Dupuis L, de Aguilar JL G, Echaniz-Laguna A (2009). Muscle mitochondrial uncoupling dismantles neuromuscular junction and triggers distal degeneration of motor neurons. PLoS One.

[10] Dupuis L, Loeffler JP (2009). Neuromuscular junction destruction during amyotrophic lateral sclerosis: insights from transgenic models. Curr Opin Pharmacol.

[11] Woodruff TM, Costantini KJ, Crane JW (2008). The complement factor C5a contributes to pathology in a rat model of amyotrophic lateral sclerosis. J Immunol.

[12] Ricklin D, Hajishengallis G, Yang K, Lambris JD (2010). Complement: a key system for immune surveillance and homeostasis. Nat Immunol.

[13] Leslie M (2012). Immunology. The new view of complement. Science.

[14] de Cordoba SR, Tortajada A, Harris CL, Morgan BP (2012). Complement dysregulation and disease: from genes and proteins to diagnostics and drugs. Immunobiology.

[15] Sta M, Sylva-Steenland RM, Casula M (2011). Innate and adaptive immunity in amyotrophic lateral sclerosis: evidence of complement activation. Neurobiol Dis.

[16] Ferraiuolo L, Heath PR, Holden H, Kasher P, Kirby J, Shaw PJ (2007). Microarray analysis of the cellular pathways involved in the adaptation to and progression of motor neuron injury in the SOD1 G93A mouse model of familial ALS. J Neurosci.

[17] Lobsiger CS, Boillee S, Cleveland DW (2007). Toxicity from different SOD1 mutants dysregulates the complement system and the neuronal regenerative response in ALS motor neurons. Proc Natl Acad Sci U S A.

[18] Humayun S, Gohar M, Volkening K (2009). The complement factor C5a receptor is upregulated in NFL-/- mouse motor neurons. J Neuroimmunol.

[19] Lee JD, Kamaruzaman NA, Fung JN (2013). Dysregulation of the complement cascade in the hSOD1G93A transgenic mouse model of amyotrophic lateral sclerosis. J Neuroinflammation.

[20] Heurich B, El Idrissi NB, Donev RM (2011). Complement upregulation and activation on motor neurons and neuromuscular junction in the SOD1 G93A mouse model of familial amyotrophic lateral sclerosis. J Neuroimmunol.

[21] Eisen A, Weber M (2001). The motor cortex and amyotrophic lateral sclerosis. Muscle Nerve.

[22] Karlsborg M, Rosenbaum S, Wiegell M (2004). Corticospinal tract degeneration and possible pathogenesis in ALS evaluated by MR diffusion tensor imaging. Amyotroph Lateral Scler Other Motor Neuron Disord.

[23] Fischer LR, Culver DG, Tennant P (2004). Amyotrophic lateral sclerosis is a distal axonopathy: evidence in mice and man. Exp Neurol.

[24] LEHRER GM, ORNSTEIN L (1959). A diazo coupling method for the electron microscopic localization of cholinesterase. J Biophys Biochem Cytol.

[25] Lin F, Fukuoka Y, Spicer A (2001). Tissue distribution of products of the mouse decay-accelerating factor (DAF) genes. Exploitation of a Daf1 knock-out mouse and site-specific monoclonal antibodies. Immunology.

[26] Liszewski MK, Farries TC, Lublin DM, Rooney IA, Atkinson JP (1996). Control of the complement system. Adv Immunol.

[27] Stahel PF, Flierl MA, Morgan BP (2009). Absence of the complement regulatory molecule CD59a leads to exacerbated neuropathology after traumatic brain injury in mice. J Neuroinflammation.

[28] Leinhase I, Holers VM, Thurman JM (2006). Reduced neuronal cell death after experimental brain injury in mice lacking a functional alternative pathway of complement activation. BMC Neurosci.

[29] Rancan M, Morganti-Kossmann MC, Barnum SR (2003). Central nervous system-targeted complement inhibition mediates neuroprotection after closed head injury in transgenic mice. J Cereb Blood Flow Metab.

[30] Anderson AJ, Robert S, Huang W, Young W, Cotman CW (2004). Activation of complement pathways after contusion-induced spinal cord injury. J Neurotrauma.

[31] Bonifati DM, Kishore U (2007). Role of complement in neurodegeneration and neuroinflammation. Mol Immunol.

[32] Ramaglia V, Wolterman R, de Kok M (2008). Soluble complement receptor 1 protects the peripheral nerve from early axon loss after injury. Am J Pathol.

[33] Ramaglia V, Tannemaat MR, de Kok M (2009). Complement inhibition accelerates regeneration in a model of peripheral nerve injury. Mol Immunol.

[34] Fluiter K, Opperhuizen AL, Morgan BP, Baas F, Ramaglia V (2014). Inhibition of the membrane attack complex of the complement system reduces secondary neuroaxonal loss and promotes neurologic recovery after traumatic brain injury in mice. J Immunol.

[35] Woodruff TM, Lee JD, Noakes PG (2014). Role for terminal complement activation in amyotrophic lateral sclerosis disease progression. Proc Natl Acad Sci USA.

[36] Lobsiger CS, Cleveland DW (2014). Reply to Woodruff et al.: C1q and C3-dependent complement pathway activation does not contribute to disease in SOD1 mutant ALS mice. Proc Natl Acad Sci U S A.

[37] Pagani MR, Reisin RC, Uchitel OD (2006). Calcium signaling pathways mediating synaptic potentiation triggered by amyotrophic lateral sclerosis IgG in motor nerve terminals. J Neurosci.

[38] Uchitel OD, Appel SH, Crawford F, Sczcupak L (1988). Immunoglobulins from amyotrophic lateral sclerosis patients enhance spontaneous transmitter release from motor-nerve terminals. Proc Natl Acad Sci U S A.

[39] Uchitel OD, Scornik F, Protti DA, Fumberg CG, Alvarez V, Appel SH (1992). Long-term neuromuscular dysfunction produced by passive transfer of amyotrophic lateral sclerosis immunoglobulins. Neurology.

[40] Appel SH, Engelhardt JI, Garcia J, Stefani E (1991). Autoimmunity and ALS: a comparison of animal models of immune-mediated motor neuron destruction and human ALS. Adv Neurol.

[41] O’Shaughnessy TJ, Yan H, Kim J (1998). Amyotrophic lateral sclerosis: serum factors enhance spontaneous and evoked transmitter release at the neuromuscular junction. Muscle Nerve.

[42] Mohamed HA, Mosier DR, Zou LL (2002). Immunoglobulin Fc gamma receptor promotes immunoglobulin uptake, immunoglobulin-mediated calcium increase, and neurotransmitter release in motor neurons. J Neurosci Res.

[43] Muchnik S, Losavio A, De LS (2002). Effect of amyotrophic lateral sclerosis serum on calcium channels related to spontaneous acetylcholine release. Clin Neurophysiol.

[44] Lobsiger CS, Boillee S, Pozniak C (2013). C1q induction and global complement pathway activation do not contribute to ALS toxicity in mutant SOD1 mice. Proc Natl Acad Sci U S A.

[45] Huber-Lang M, Sarma JV, Zetoune FS (2006). Generation of C5a in the absence of C3: a new complement activation pathway. Nat Med.

[46] Mantovani S, Gordon R, Macmaw JK (2014). Elevation of the terminal complement activation products C5a and C5b-9 in ALS patient blood. J Neuroimmunol.

[47] Bahia El Idrissi N, Das PK, Fluiter K, et al. M. leprae components induce nerve damage by complement activation: identification of lipoarabinomannan as the dominant complement activator. Acta Neuropathol. 2015;129:653–67. 10.1007/s00401-015-1404-5 PMC4405335 25772973

[48] Walport MJ (2001). Complement. First of two parts. N Engl J Med.

[49] Walport MJ (2001). Complement. Second of two parts. N Engl J Med.

[50] Soltys J, Halperin JA, Xuebin Q (2012). DAF/CD55 and Protectin/CD59 modulate adaptive immunity and disease outcome in experimental autoimmune myasthenia gravis. J Neuroimmunol.

