# Supplementary figures and images for: Complement activation at the motor end-plates in amyotrophic lateral sclerosis
Source: J Neuroinflammation. 2016 Apr 7;13:72. doi: 10.1186/s12974-016-0538-2 (PMC4823861; doi:10.1186/s12974-016-0538-2)

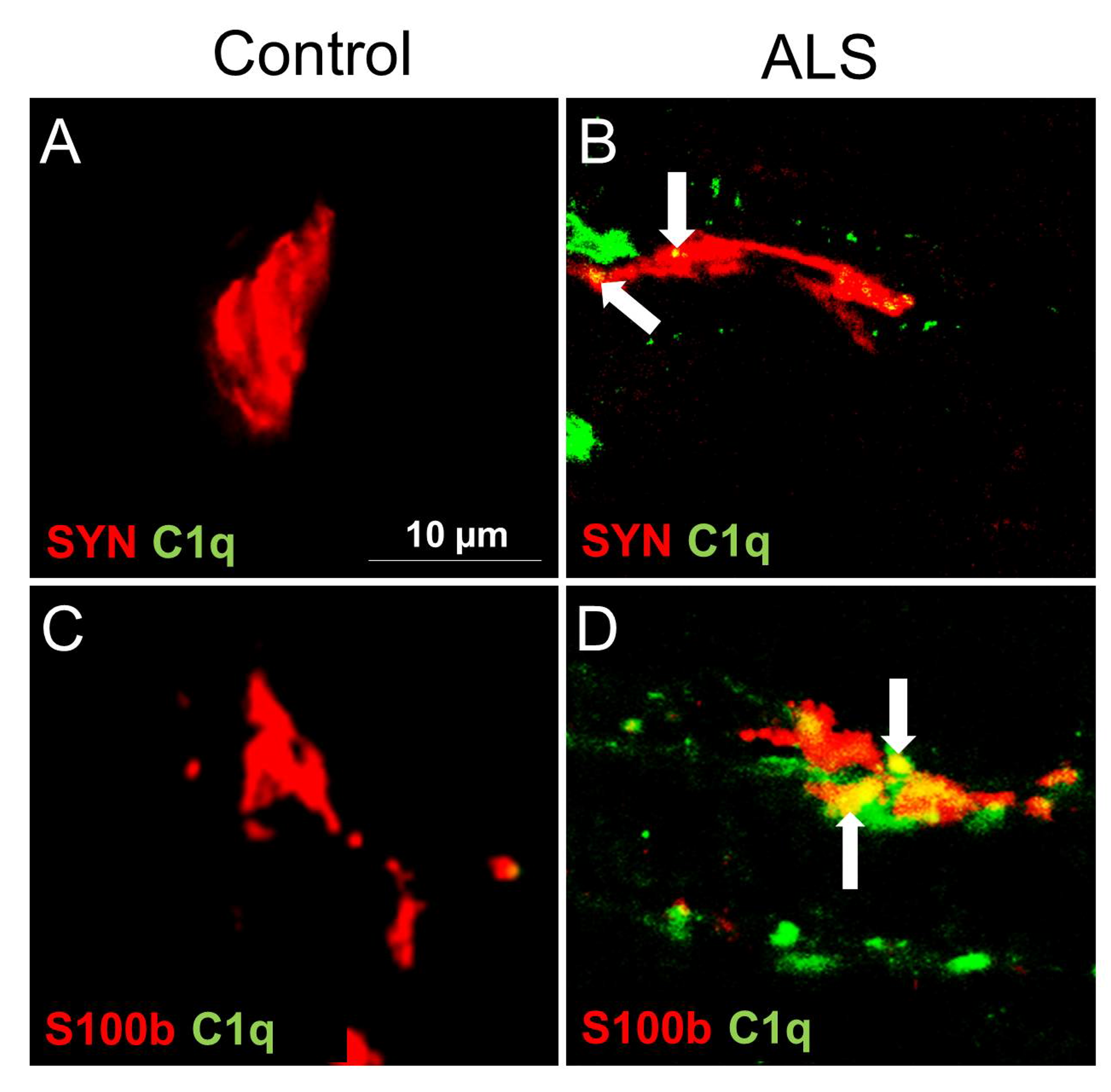

Supplement: Additional file 1: Figure S1. — Representative confocal immunofluorescence for synaptophysin (SYN-Cy3) detecting the motor nerve terminal (A, B) or S100b (Cy3) detecting the terminal Schwann cells (C, D) double stained with anti-C1q (FITC) in control (A, C) and ALS (B, D) intercostal muscle shows C1q co-localizing with both synaptophysin and S100b (white arrow in B and D, respectively) but no C1q deposition in controls. (TIF 1461 kb) [file 12974_2016_538_MOESM1_ESM.tif]

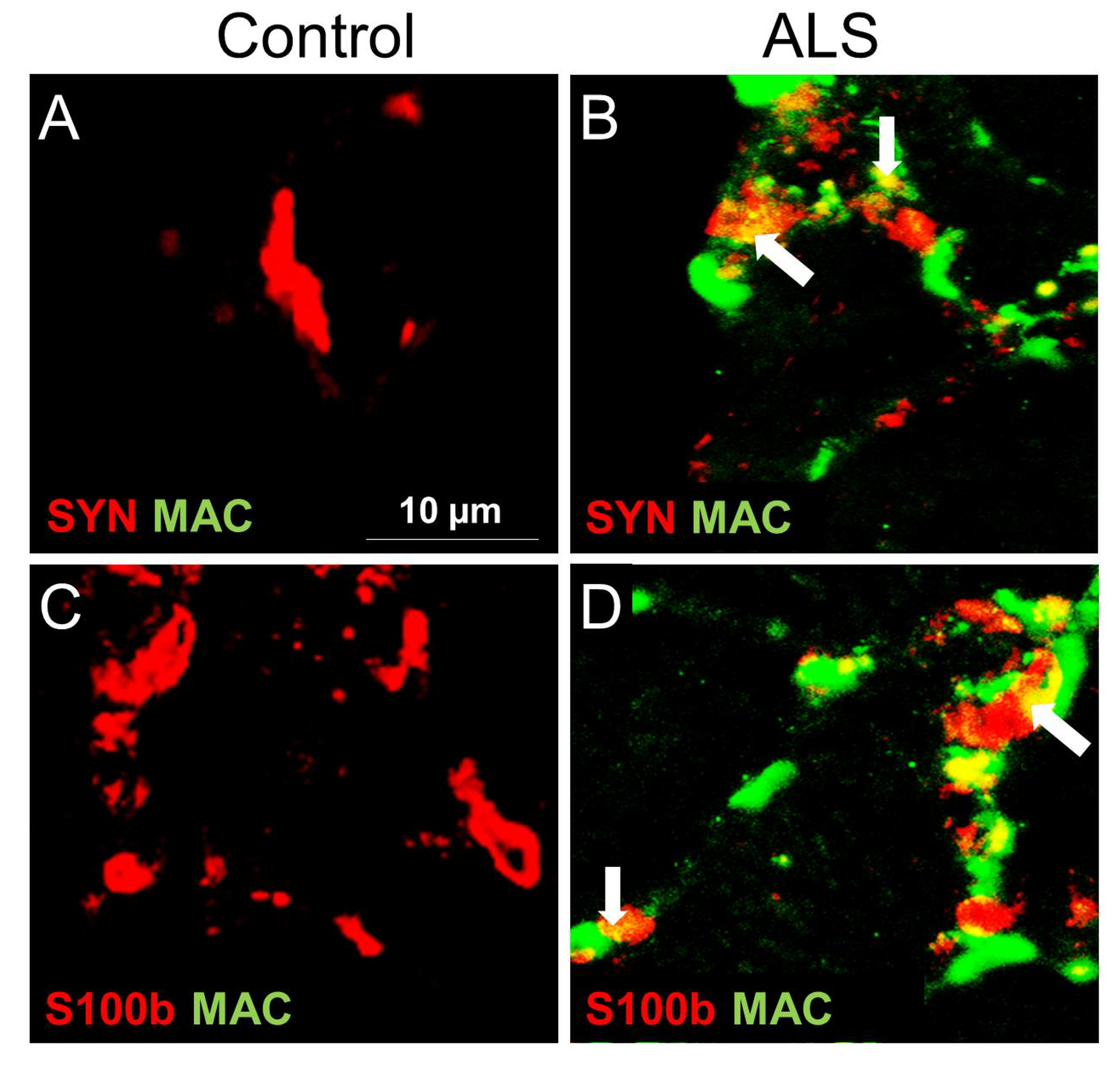

Supplement: Additional file 2: Figure S2. — Representative confocal immunofluorescence for synaptophysin (SYN-Cy3) detecting the motor nerve terminal (A, B) or S100b (Cy3) detecting the terminal Schwann cells (C, D) double stained with an antibody detecting MAC (FITC) in control (A, C) and ALS (B, D) intercostal muscle shows MAC deposition on both the motor nerve terminal and the terminal Schwann cells (white arrow in B and D, respectively) but no MAC deposition in controls. (TIF 2178 kb) [file 12974_2016_538_MOESM2_ESM.tif]

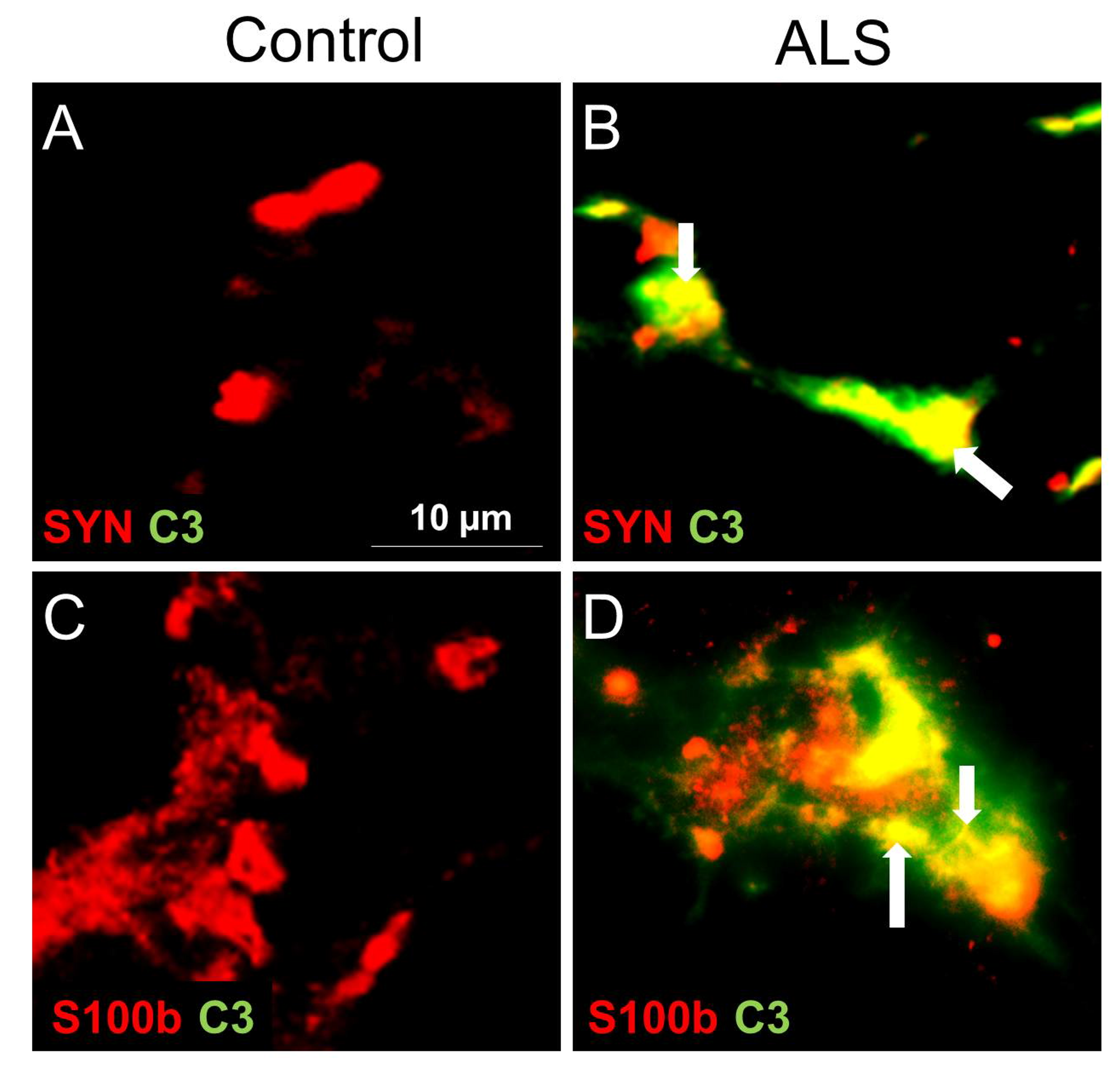

Supplement: Additional file 3: Figure S3. — Representative confocal immunofluorescence for synaptophysin (SYN-Cy3) detecting the motor nerve terminal (A, B) or S100b (Cy3) detecting the terminal Schwann cells (C, D) double stained with anti-C3c recognizing C3c part of C3 and C3b (FITC) in control (A, C) and ALS (B, D) intercostal muscle shows C3c co-localizing with both synaptophysin and S100b (white arrow in B and D, respectively), but no C3c deposition in controls. (TIF 1624 kb) [file 12974_2016_538_MOESM3_ESM.tif]

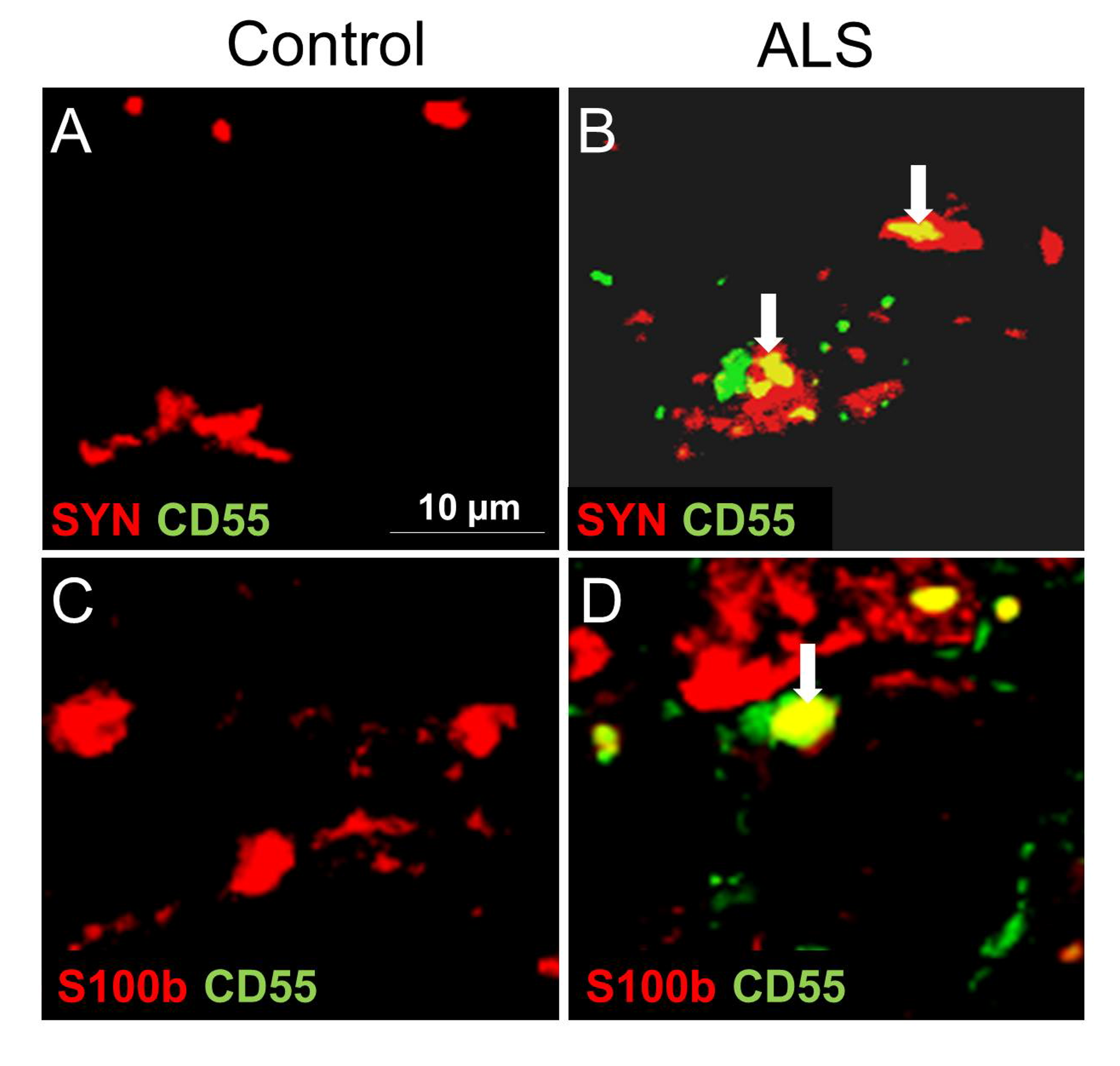

Supplement: Additional file 4: Figure S4. — Representative confocal immunofluorescence for synaptophysin (SYN-Cy3) detecting the motor nerve terminal (A, B) or S100b (Cy3) detecting the terminal Schwann cells (C, D) double stained with anti-CD55 (FITC) in control (A, C) and ALS (B, D) intercostal muscle shows CD55 co-localizing with both synaptophysin and S100b (white arrow in B and D, respectively) but no CD55 deposition in controls. (TIF 1271 kb) [file 12974_2016_538_MOESM4_ESM.tif]

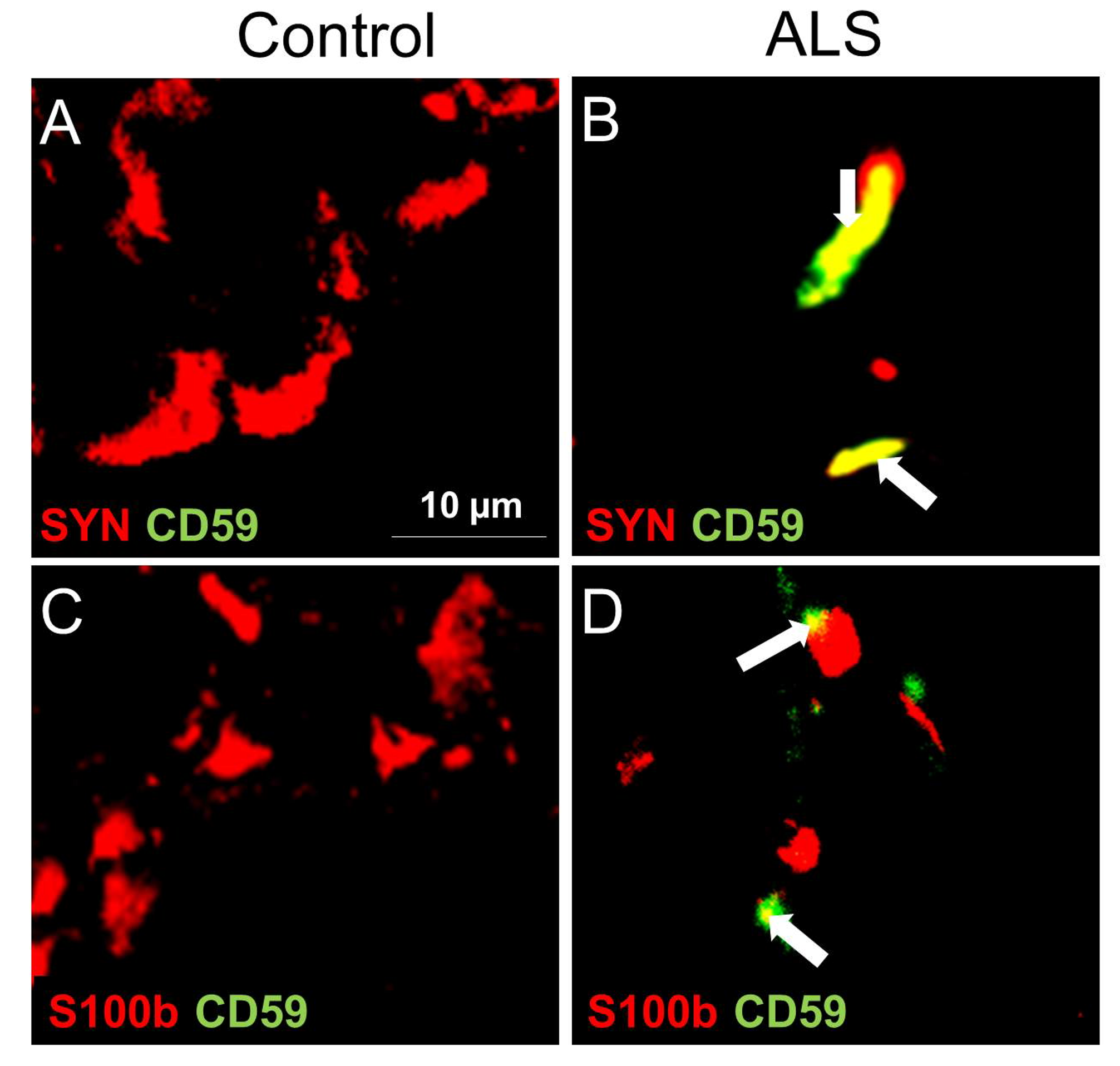

Supplement: Additional file 5: Figure S5. — Representative confocal immunofluorescence for synaptophysin (SYN-Cy3) detecting the motor nerve terminal (A, B) or S100b (Cy3) detecting the terminal Schwann cells (C, D) double stained with anti-CD59 (FITC) in control (A, C) and ALS (B, D) intercostal muscle shows CD59 deposition on both the motor nerve terminal and the terminal Schwann cells (white arrow in B and D, respectively) but no CD59 deposition in controls. (TIF 1202 kb) [file 12974_2016_538_MOESM5_ESM.tif]

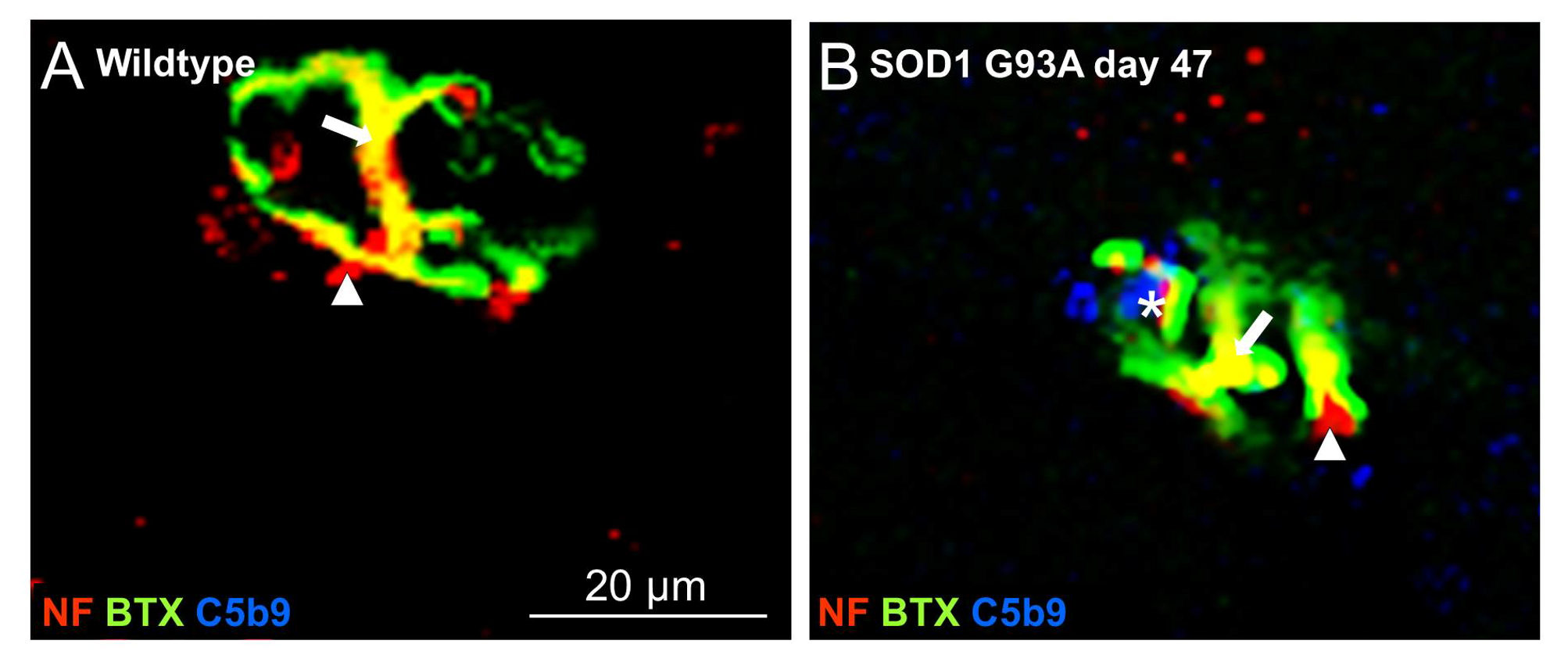

Supplement: Additional file 6: Figure S6. — Representative confocal microscopy images of the motor end-plate from wild-type (n = 4) (A) and SOD1G93A mice (n = 4) at 47 (B), immunostained for neurofilament NF-H (white arrow head in A and B), MAC with C5b9 (white asterisk in B), and the muscle end-plate with α-BTX (Alexa 488), showing deposition of MAC (white asterisk in B) on the innervated motor end-plate (white arrow pointing to NF-H co-localizing with α-BTX) in SOD1G93A mice but not in the wild-type mice. Bar = 20 μm. (TIF 869 kb) [file 12974_2016_538_MOESM6_ESM.tif]
